# Multi-dimensional sleep health and dementia risk: a prospective study in the UK Biobank

**DOI:** 10.1186/s12916-025-04251-3

**Published:** 2025-07-07

**Authors:** Tianyi Huang, May A. Beydoun, Sina Kianersi, Susan Redline, Lenore J. Launer

**Affiliations:** 1 Laboratory of Epidemiology and Population Sciences, Intramural Research Program, National Institute On Aging, Baltimore, MD USA; 2 Division of Sleep and Circadian Disorders, Department of Medicine, Brigham and Women’s Hospital, Boston, MA USA; 3 Division of Sleep Medicine, Harvard Medical School, Boston, MA USA

**Keywords:** Alzheimer’s disease, Dementia, Health promotion, Latent class analysis, Prevention, Prospective study, Sleep, Sleep health, Vascular dementia

## Abstract

**Background:**

The intricate interplay of various sleep characteristics may influence dementia risk through different pathogenic pathways. However, few studies have examined multi-dimensional sleep health in relation to dementia risk or explored potential etiologic heterogeneity by dementia subtypes.

**Methods:**

Our study included 313,248 UK Biobank participants aged ≥ 50 years who were dementia-free in 2006–2010. Incident dementia was identified using validated algorithms through primary care, hospital admissions, or death records through 2022. Multi-dimensional sleep health was evaluated based on seven self-reported sleep-related factors and assessed in two ways: (1) using an a priori sleep health score (SHS) ranging from 0 to 7, with higher scores indicating healthier sleep, and (2) through data-driven sleep health patterns identified by latent class analysis. We used Cox proportional hazards models to estimate the associations between multi-dimensional sleep health and risk of all-cause dementia, vascular dementia (VaD), and Alzheimer’s disease (AD).

**Results:**

There were 7458 incident all-cause dementia cases (1636 VaD, 3376 AD) after 4,165,352 person-years of follow-up. After adjusting for potential confounders, the hazard ratio (95% CI) comparing participants with SHS of 0–2 (worst sleep) vs 6–7 (best sleep) was 1.76 (1.52, 2.05) for all-cause dementia (*p*-trend < 0.0001), 2.13 (1.61, 2.83) for VaD (*p*-trend < 0.0001), and 1.55 (1.22, 1.97) for AD (*p*-trend < 0.57). We identified six multi-dimensional sleep health patterns, including relatively healthy sleep, insomnia with short sleep duration, non-restorative sleep with evening chronotype, insomnia with non-restorative sleep, snoring with daytime sleepiness and napping, and severely disturbed sleep with multiple symptoms and daytime impairment. Compared with the healthy sleep pattern, all other five sleep patterns were significantly associated with 8–85% higher all-cause dementia risk and 11–148% higher VaD risk, whereas only the severely disturbed sleep pattern was associated with 56% higher AD risk (95% CI: 1.21, 2.01).

**Conclusions:**

Poor multi-dimensional sleep health, either assessed by a simple SHS or characterized by sleep clusters, was associated with higher incident dementia risk. There is substantial heterogeneity in multi-dimensional sleep health patterns and their associations with different dementia outcomes. Understanding the specific sleep health profiles associated with dementia risk may help to identify high-risk populations and inform more targeted interventions.

**Supplementary information:**

The online version contains supplementary material available at 10.1186/s12916-025-04251-3.

## Background

A growing body of evidence supports the vital role of sleep health in older populations, demonstrating its positive impact on improving cognitive functions, mitigating dementia risk, and fostering overall healthy aging. Previous studies have linked sleep duration, sleep quality, and sleep-related traits (e.g., chronotype), along with sleep disorders and related sleep disturbances and daytime symptoms, to dementia risk [1–8]. However, a major limitation of these studies is that sleep health factors have typically been examined individually in relation to dementia risk, overlooking the co-occurrence and potential interactions of multiple sleep phenotypes. A more holistic approach is needed to fully understand the complex relationship between sleep health and dementia risk.

The multi-dimensional sleep health framework is proposed to address the fact that individual sleep health factors are rarely present in isolation [9, 10]. Instead, certain sleep factors often cluster together, forming distinct sleep phenotypes with significant clinical implications [11, 12]. For example, insomnia is a highly heterogeneous disorder [13–16]. Specifically, insomnia with objective short sleep duration is considered a more biologically severe phenotype associated with adverse cardiometabolic and neurocognitive outcomes [13, 14]. Co-morbid insomnia and sleep apnea (COMISA) represents a stronger risk factor for diabetes and cardiovascular disease than either disorder alone, posing clinical challenges in co-managing both disorders [15, 16]. Further, insomnia may result from circadian misalignment, be secondary to co-morbid conditions (e.g., chronic pain), or occur episodically in response to stressful life events. Therefore, examining the association between a sleep health factor and dementia risk without considering other co-occurring sleep dimensions would fail to capture the underlying etiologic complexity and the true health impact of the sleep problems, potentially leading to misleading conclusions.

There are established age and sex differences in sleep health. Sleep duration tends to decline with age [17], insomnia symptoms are more prevalent in women [18], and sleep apnea is more common in men [19]. However, it remains inconclusive how age and sex may modify the associations between sleep health and dementia risk. As a potentially modifiable lifestyle factor, sleep health may interact with genetic susceptibility to exacerbate or mitigate dementia risk. Yet, these gene-sleep interactions have not been fully elucidated, especially in the context of multi-dimensional sleep health. In the present study, we aimed to derive sleep health patterns under the multi-dimensional framework and evaluate how different sleep health patterns may relate to incident dementia risk in the UK Biobank. Multi-dimensional sleep health was assessed using an a priori sleep health score (SHS) and through data-driven sleep health patterns based on seven self-reported factors. We hypothesized that a greater number of unhealthy sleep factors would be more strongly associated with an increased risk of dementia and that this association would vary across the distinct sleep health patterns identified.

## Methods

### Study population

The UK Biobank is a large-scale cohort study of over 500,000 UK adults aged 37–73 years at recruitment that aims to improve the prevention, diagnosis, and treatment of a wide range of diseases. At baseline (2006–2010), participants completed comprehensive questionnaires (including sleep health), underwent physical measurements, and provided biological samples for biochemical assays and genotyping. Disease occurrence was prospectively identified from linked healthcare data. Our study focused on 384,940 participants aged ≥ 50 years at baseline. After excluding participants with missing or invalid information on any sleep health factors (*n* = 71,531) or with prevalent dementia (*n* = 161), 313,248 were included in the analysis.

### Sleep assessment

Participants completed a touch-screen questionnaire that assessed their sleep health in seven important dimensions: (1) sleep duration, (2) insomnia symptoms, (3) non-restorative sleep, (4) snoring, (5) daytime sleepiness, (6) napping, and (7) chronotype. The assessment questions and the detailed categorization of responses are presented in Additional file 1: Table S1. Briefly, after excluding participants reporting < 3 h or > 14 h of sleep, we categorized sleep duration was short (< 7 h), medium (7–8 h), and long (> 8 h) to allow potential nonlinear relationships with dementia outcomes. To reflect that chronotype is a gradient trait along a continuum (20), chronotype was categorized into morning (definitely a “morning” person), intermediate (more a “morning” than “evening” person, or more an “evening” than “morning” person), and evening (definitely an “evening” person). The remaining five factors were assessed as binary variables. As sleep disturbances are common in older populations, participants who reported “sometimes” experiencing insomnia, daytime sleepiness, or napping were combined with those who reported “never/rarely.” This categorization helped focus on more clinically relevant sleep patterns while minimizing potential misclassification from short-term, episodic sleep disturbances.

The SHS was defined a priori, with 1 point assigned for meeting each of the seven criteria and 0 assigned otherwise: 7–8 h of sleep duration, absence of frequent insomnia symptoms, daytime sleepiness, or napping, no self-reported snoring, restorative sleep, and an intermediate chronotype. The SHS ranged from 0 to 7, with higher scores indicating healthier sleep. Based on the distribution, the SHS was further categorized into four groups representing minimal (6–7), mild (5), moderate (3–4), and severe (0–2) sleep disturbances.

To evaluate the stability of baseline self-reported sleep measurements, we calculated the intraclass correlation coefficient (ICC) for SHS and Cohen’s kappa for individual sleep factors among two subsets of participants. The first subset consisted of 12,010 participants who repeated the sleep assessment in 2009–2013, and the second included 3318 participants who repeated the assessment in 2019–2023. For the first group, the ICC for SHS (measured an average of 4.2 years apart) was 0.58, with Cohen’s kappa ranging from 0.35 for sleepiness to 0.65 for snoring. In contrast, for the second group (with assessments 12.2 years apart), the ICC for SHS was 0.43, and kappa values ranged from 0.22 for sleepiness to 0.53 for snoring. Overall, self-reported sleep health remains modestly stable over time in older adults, even over a span of more than a decade.

### Dementia ascertainment

Dementia cases were identified through linkage to primary care, hospital admissions, or death records using a validated algorithm based on the International Classification of Diseases 10th revision (ICD-10) codes (21). Self-reported history at baseline was incorporated to identify prevalent dementia. The outcomes of interest included incident all-cause dementia and its subtypes of Alzheimer’s disease (AD) and vascular dementia (VaD) (Additional file 1: Table S2). In a prior validation study of 120 UK Biobank participants with ≥ 1 dementia codes from health records (including 63 with AD codes and 16 with VaD codes), this algorithm demonstrated a positive predicted value (PPV) of 82.5% for identifying all-cause dementia, with lower PPVs for AD (71.4%) and VaD (43.8%) [20].

### Statistical analysis

We used line plots to show the age trends of each sleep health factor and the SHS, separately for men and women. We calculated means and standard deviations for continuous variables and percentages for categorical variables in the overall sample and across the SHS categories. Cox proportional hazards models were used to estimate the hazard ratios (HRs) and 95% CI for the associations between the SHS categories (SHS of 6–7 as the reference) and incident dementia risk. To account for varying baseline hazards of dementia across different ages, we used age as the timescale in the Cox models. Each participant contributed person-time from the age at which they completed the baseline sleep assessment until the earliest age at which any of the following events occurred: dementia diagnosis, death, loss to follow-up, or the censoring date (December 31, 2022). We also estimated Kaplan–Meier curves with age as the timescale accounting for left truncation and right censoring. No significant deviations from the proportional hazards assumption were detected based on the Schoenfeld residuals. We considered two primary multivariable models: model 1 adjusted for age in months, sex, race and ethnicity, Townsend deprivation index, education in years, employment status, and family history of dementia, and model 2 further adjusted for smoking status, alcohol consumption, physical activity, and body mass index. As a sensitivity analysis, we additionally added to model 3 total cholesterol, HDL cholesterol, systolic blood pressure, diastolic blood pressure, HbA1c, and depression, which may be intermediate pathways linking sleep health to dementia development. All analyses were conducted for all-cause dementia as well as for VaD and AD separately. The linear trend was tested by modeling the SHS as a continuous variable.

To address potential reverse causation, we excluded dementia cases diagnosed in the first 5 years of follow-up and repeated the primary analysis. We performed subgroup analyses to examine whether the associations of the SHS with risks of all-cause dementia and dementia subtypes differed by age (≥ 60, < 60 years), sex (men, women), and the polygenic risk score (PRS) for AD (in tertiles). Statistical significance of the subgroup difference was assessed using a likelihood ratio test, comparing models with and without the multiplicative interaction term between the continuous SHS and the subgroup factor.

Next, we used latent class analysis (LCA) to group participants into clusters that shared a similar sleep health pattern using the R package *poLCA* [21, 22]. LCA is a data-driven approach that identifies unobserved subgroups based on observed response patterns and estimated group membership probabilities using the Expectation–Maximization algorithm. Given the large sample size, we determined the optimal number of clusters based on interpretability and to prevent overfitting, ensuring that the smallest cluster contained > 1% of the total sample. We also calculated the posterior predicted probability for each participant of belonging to the assigned cluster as a measure of model fit. The distribution of the sleep health factors within each cluster was visualized using a spider plot. We conducted a similar prospective analysis using Cox models to estimate dementia risk according to identified sleep health clusters, adjusted for covariates in model 2 as outlined above.

We performed additional secondary analyses to gain deeper insight into the observed associations. First, to further explore the clinical relevance of the derived multi-dimensional sleep health patterns, we examined both cross-sectional and prospective associations of SHS and sleep health clusters with clinically diagnosed sleep disorders, as defined by ICD-10 code G47. Second, to facilitate comparison with previous studies (which did not restrict their analytical samples to those aged 50 years or older) [4–6], we also assessed the associations between individual sleep health risk factors and dementia risk using multivariable Cox models. Third, since poor sleep health may increase risk of premature death prior to dementia onset potentially leading to biased interpretations [23], we conducted competing risks Cox analysis using the R package *cmprsk* to account for non-dementia-related mortality as a competing event [24]. All analyses were performed using RStudio (version 4.4.0) on the UK Biobank Research Analysis Platform.

## Results

The mean age at baseline was 60.5 years (SD: 5.5), with 45.2% of participants being men and 96.0% identifying as white (Table 1). Participants with poorer sleep health were more likely to be men, non-white, socioeconomically disadvantaged, less educated, and either unemployed or unable to work, compared with those with the best sleep health (SHS 6–7 points). Lower SHS was associated with current/former smoking, reduced alcohol consumption, lower physical activity, higher BMI and HbA1c, lower total and HDL cholesterol, and greater prevalence of depression. Family history of dementia and PRS for AD did not differ appreciably across SHS categories.

**Table 1:** Baseline characteristics across multi-dimensional sleep health score categories among participants aged 50 years or older without clinically diagnosed dementia in the UK Biobank (2006–2010)

	Overall	Multi-dimensional sleep health score			
		6–7 (best sleep)	5	3–4	0–2 (worst sleep)
*N*	313,248	158,057	87,942	63,068	4181
Age, years	60.5 (5.5)	60.6 (5.5)	60.5 (5.4)	60.4 (5.4)	60.5 (5.5)
Men, %	45.2	44.0	46.8	45.5	53.0
White, %	96.0	96.7	95.7	94.9	92.5
Townsend deprivation index	− 1.5 (3.0)	− 1.7 (2.8)	− 1.5 (3.0)	− 1.2 (3.1)	− 0.3 (3.4)
Education, years	14.6 (5.2)	14.9 (5.1)	14.4 (5.2)	14.1 (5.3)	13.4 (5.4)
Employment status					
Employed	48.9	49.7	50.0	46.7	33.3
Retired	43.2	44.1	42.4	42.1	43.3
Unemployed/unable to work	7.8	6.2	7.6	11.3	23.4
Family history of dementia, %	13.8	13.7	13.7	14.0	13.5
Smoking status, %					
Never	52.6	55.2	51.4	48.6	41.0
Current	9.3	8.0	9.6	11.7	17.5
Former	38.0	36.7	39.0	39.7	41.6
Alcohol drinking, %					
Never/special occasion only	18.9	16.9	19.2	22.8	32.1
1–3 per month	10.2	10.1	10.0	10.8	11.1
1–4 per week	48.5	50.6	47.9	44.6	38.3
Daily/almost daily	22.3	22.4	22.9	21.7	18.4
Physical activity, MET-hours/week	44.4 (44.0)	45.4 (43.3)	44.4 (44.5)	42.6 (45.4)	33.9 (42.2)
Body mass index, kg/m^2^	27.5 (4.7)	26.9 (4.3)	27.7 (4.7)	28.6 (5.1)	30.7 (6.1)
Systolic blood pressure, mmHg	140.4 (18.7)	140.4 (18.8)	140.6 (18.6)	140.3 (18.5)	139.7 (18.4)
Diastolic blood pressure, mmHg	82.6 (10.0)	82.3 (10.0)	82.7 (10.0)	82.9 (10.1)	83.1 (10.4)
Total cholesterol, mg/dL	222.9 (45.3)	224.6 (44.4)	222.3 (45.4)	220.3 (46.6)	211.9 (49.4)
HDL cholesterol, mg/dL	56.6 (15.1)	57.7 (15.1)	56.2 (14.9)	54.9 (14.8)	50.6 (13.9)
HbA1c, mmol/mol	36.7 (6.8)	36.2 (6.1)	36.8 (7.0)	37.4 (7.6)	39.9 (10.5)
Depression^a^, %	36.9	31.6	37.8	47.3	63.2
Polygenic risk score for AD	0.05 (1.00)	0.06 (1.00)	0.05 (0.99)	0.04 (1.00)	0.03 (0.97)
Sleep health					
Sleep duration, %					
Short (< 7 h)	24.4	6.6	31.5	56.9	60.5
Medium (7–8 h)	67.3	90.1	57.6	27.5	9.7
Long (> 8 h)	8.3	3.3	10.8	15.6	29.8
Insomnia symptoms, %	29.8	8.9	37.2	67.7	88.5
Non-restorative sleep, %	15.3	4.8	17.2	35.5	68.6
Snoring, %	38.3	21.9	49.1	61.6	82.9
Daytime sleepiness, %	2.9	0.3	1.7	8.0	52.2
Napping, %	6.0	1.2	5.5	15.4	57.4
Chronotype, %					
Morning	28.5	16.2	37.9	45.6	39.6
Intermediate	63.4	81.5	53.1	35.4	19.3
Evening	8.0	2.3	9.0	19.0	41.1

^a^Based on a broad depression definition if one or more of the following was met: Patient Health Questionnaire-2 (PHQ-2) score ≥ 3 or self-reported clinical visits for depression

### Trends in multi-dimensional sleep health by age and sex

**Fig. 1:**
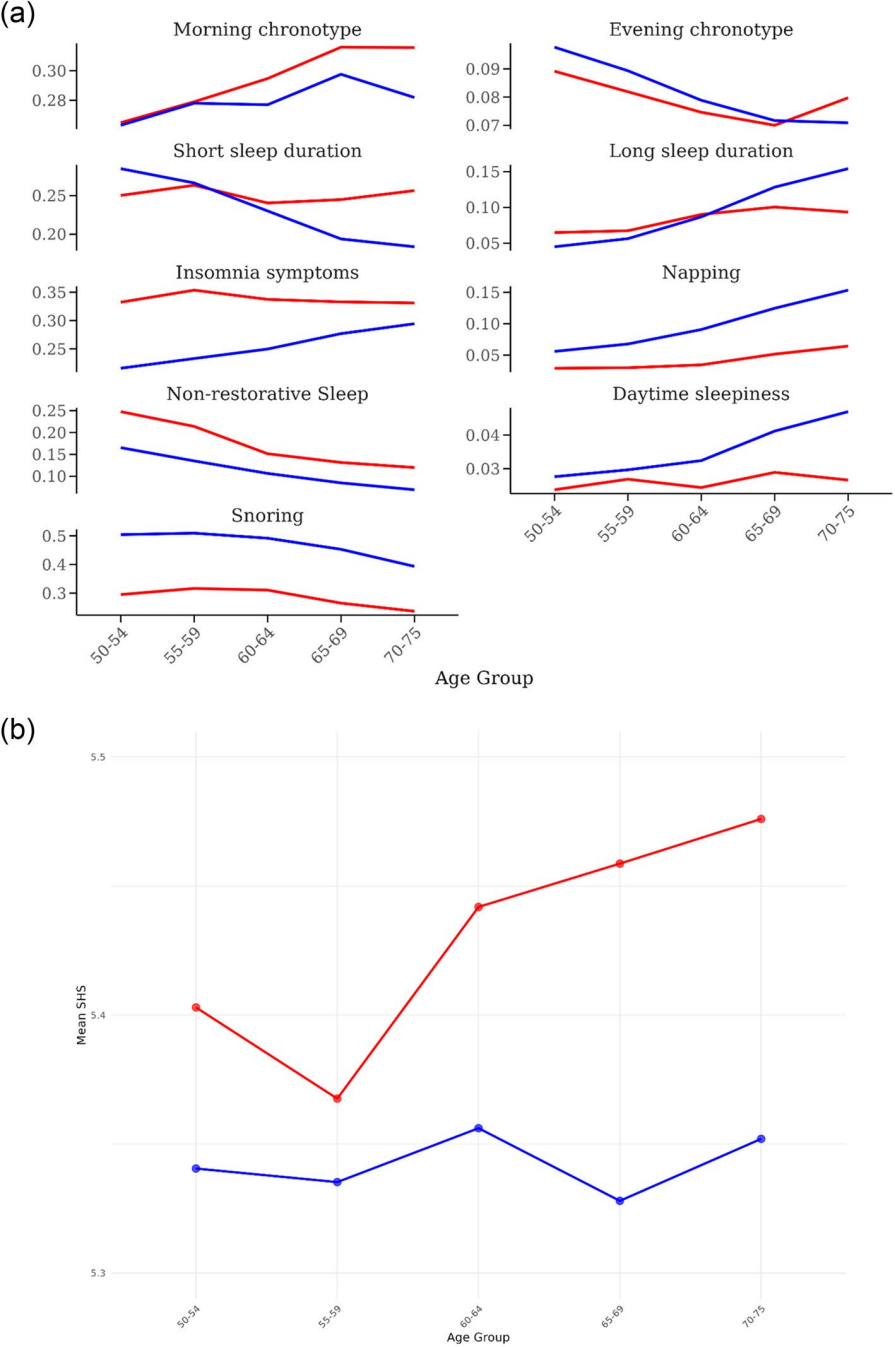
Age trends of multi-dimensional sleep health in men (blue) and women (red) in the UK Biobank (2006–2010). **a** Individual sleep health factors. **b** The sleep health score (SHS). Note that the y-axis for SHS is scaled from 5.3 to 5.5, although the full SHS scale ranges from 0 to 7

We observed an age-related increase in self-reported morning chronotype and a decrease in evening chronotype, with similar patterns by sex (Fig. 1a). The overall prevalence of short and long sleep duration was similar between men and women. With increasing age, men showed a decline in short sleep and an increase in long sleep, whereas in women these prevalence estimates remained relatively stable. Insomnia symptoms and non-restorative sleep were consistently more prevalent in women than men across age groups. However, the prevalence of insomnia increased with age only in men, narrowing the sex difference in older groups. Both men and women exhibited a decline in non-restorative sleep with age. In contrast, the prevalence of snoring, daytime sleepiness, and napping was consistently higher in men than women, with men showing a more pronounced age-related increase in daytime sleepiness and napping. Snoring tended to decrease with aging in both sexes. Overall, the mean SHS was slightly higher in women than men, and the sex difference in SHS was somewhat enlarged with age (Fig. 1b).

### Sleep health score and dementia risk

After 4,165,352 person-years of follow-up, there were 7458 incident all-cause dementia cases (1636 VaD and 3376 AD; Table 2 and Additional file 1: Fig. S1). Compared with participants with SHS of 6–7 (best sleep health), the HR (95% CI) for all-cause dementia were 1.07 (1.01, 1.13) for SHS of 5, 1.18 (1.11, 1.25) for 3–4, and 1.76 (1.52, 2.05) for 0–2 (worst sleep health), after accounting for sociodemographic factors, family history of dementia, and lifestyle factors (*p* for trend < 0.0001). We observed stronger associations for VaD. The comparable HR (95% CI) across SHS categories was 1.00 (reference), 1.17 (1.04, 1.32), 1.36 (1.20, 1.54), and 2.13 (1.61, 2.83), respectively (*p* for trend < 0.0001). By contrast, increased AD risk was only observed comparing participants with SHS of 0–2 versus 6–7 (HR: 1.55; 95% CI: 1.22, 1.97). No increased risk was found in participants with SHS of 5 or 3–4 (*p* for trend = 0.57). Further adjustment for cardiovascular risk factors and depression modestly attenuated the associations but did not alter these results substantially.

**Table 2:** Associations of the multi-dimensional sleep health score with risk of all-cause dementia, vascular dementia, and Alzheimer’s disease among participants aged 50 years or older in the UK Biobank (2006–2022)

	Multi-dimensional sleep health score				*p* for trend
	6–7 (best sleep)	5	3–4	0–2 (worst sleep)	
All-cause dementia					
Cases	3463	2113	1697	185	
Person-years	2,112,240	1,168,838	831,418	52,856	
Rate per 1000 person-years	16.4	18.1	20.4	35.0	
Model 1^a^	1.00 (ref)	1.08 (1.02, 1.14)	1.21 (1.14, 1.28)	1.88 (1.62, 2.18)	< 0.0001
Model 2^b^	1.00 (ref)	1.07 (1.01, 1.13)	1.18 (1.11, 1.25)	1.76 (1.52, 2.05)	< 0.0001
Model 3^c^	1.00 (ref)	1.06 (0.99, 1.12)	1.12 (1.04, 1.20)	1.60 (1.33, 1.91)	< 0.0001
Vascular dementia					
Cases	685	478	419	54	
Person-years	2,112,240	1,168,838	831,418	52,856	
Rate per 1000 person-years	3.2	4.1	5.0	10.2	
Model 1^a^	1.00 (ref)	1.21 (1.08, 1.37)	1.47 (1.30, 1.66)	2.55 (1.93, 3.37)	< 0.0001
Model 2^b^	1.00 (ref)	1.17 (1.04, 1.32)	1.36 (1.20, 1.54)	2.13 (1.61, 2.83)	< 0.0001
Model 3^c^	1.00 (ref)	1.13 (0.99, 1.30)	1.29 (1.12, 1.50)	1.72 (1.22, 2.45)	< 0.0001
Alzheimer’s disease					
Cases	1717	913	674	72	
Person-years	2,112,240	1,168,838	831,418	52,856	
Rate per 1000 person-years	8.1	7.8	8.1	13.6	
Model 1^a^	1.00 (ref)	0.95 (0.88, 1.03)	0.98 (0.89, 1.07)	1.55 (1.22, 1.96)	0.54
Model 2^b^	1.00 (ref)	0.95 (0.88, 1.03)	0.98 (0.89, 1.07)	1.55 (1.22, 1.97)	0.57
Model 3^c^	1.00 (ref)	0.94 (0.86, 1.03)	0.91 (0.82, 1.01)	1.42 (1.07, 1.90)	0.59

^a^Adjusted for age in months, sex, race/ethnicity, Townsend deprivation index, education in years, employment status, and family history of dementia

^b^Model 1 + adjusted for smoking status, alcohol consumption, physical activity, and body mass index

^c^Model 2 + adjusted for total cholesterol, HDL cholesterol, systolic blood pressure, diastolic blood pressure, HbA1c, and depression

The association patterns remained largely the same after we excluded dementia cases diagnosed within the first 5 years of follow-up (Additional file 1: Table S3). Increased dementia risk associated with lower SHS was consistently observed across subgroups defined by age (Additional file 1: Table S4), sex (Additional file 1: Table S5), and PRS for AD (Additional file 1: Table S6). However, the associations were significantly stronger in participants < 60 years versus ≥ 60 years (*p* for interaction < 0.05) and in participants with lower versus higher genetic susceptibility to AD (*p* for interaction < 0.001). While the association estimates were generally larger in women than in men, only the sex difference in the association between SHS and VaD risk reached statistical significance (*p* for interaction = 0.03).

### Multi-dimensional sleep health patterns and dementia risk

**Fig. 2:**
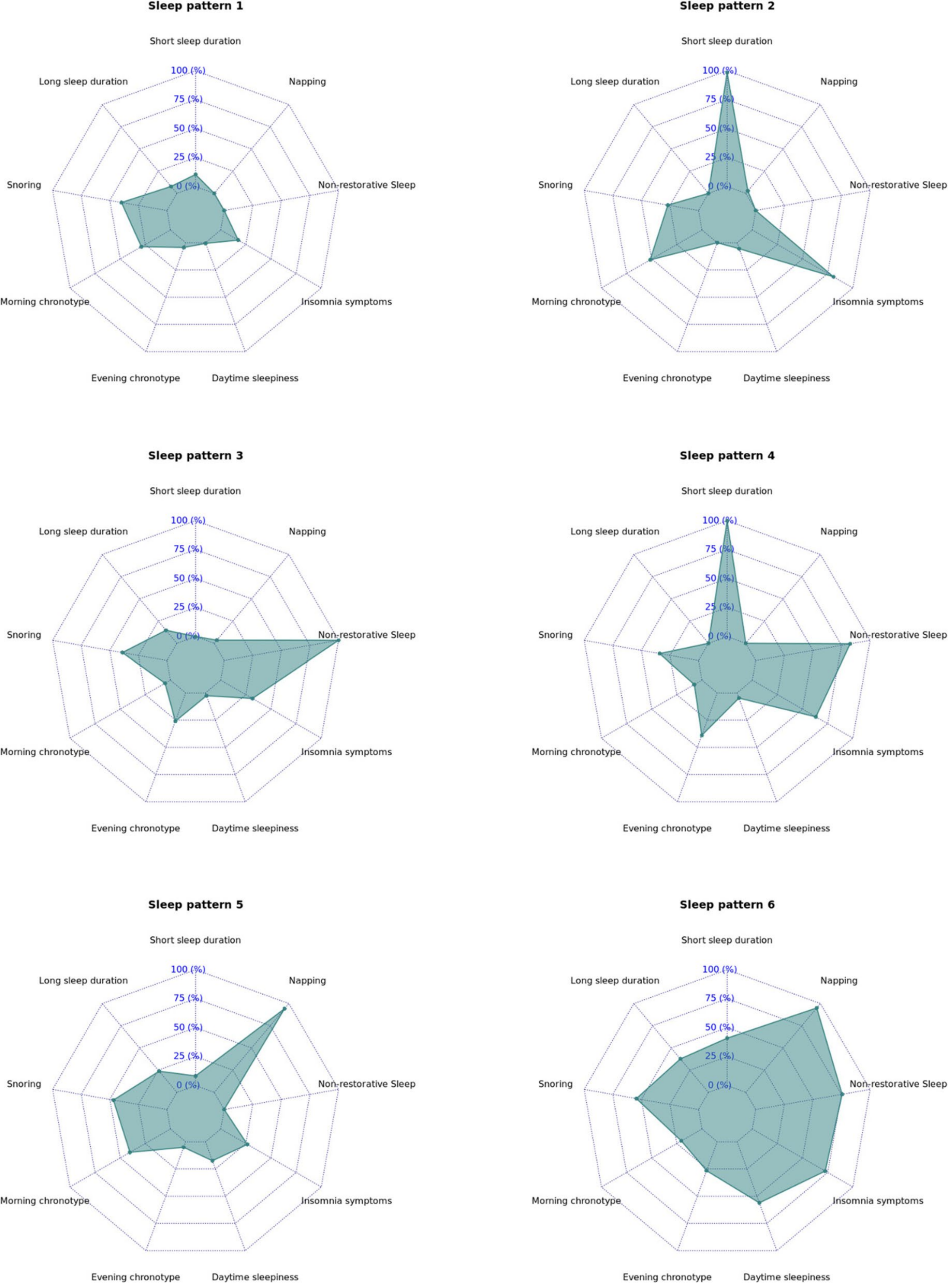
Clusters of multi-dimensional sleep health patterns identified by the latent class analysis approach in the UK Biobank (2006–2010)

The LCA model identified six multi-dimensional sleep health patterns (Fig. 2):

- Sleep pattern 1 (*N* = 209,918): relatively healthy sleep.
- Sleep pattern 2 (*N* = 37,671): insomnia (80.8%) with short sleep duration (98.4%) and morning chronotype (51.6%).
- Sleep pattern 3 (*N* = 33,112): non-restorative sleep (100%) with evening chronotype (26.0%).
- Sleep pattern 4 (*N* = 15,031): insomnia (63.3%) with short sleep duration (99.9%), non-restorative sleep (82.6%), and evening chronotype (38.9%).
- Sleep pattern 5 (*N* = 14,269): napping (94.2%) with snoring (46.9%), long sleep duration (23.8%), and daytime sleepiness (17.4%).
- Sleep pattern 6 (*N* = 3247): severely disturbed sleep with multiple symptoms and daytime impairment.

The average posterior probability was > 0.7 for all six identified clusters (0.86, 0.73, 0.86, 0.93, 0.89, 0.81, respectively), suggesting high within-cluster reliability and excellent discrimination between clusters. The distributions of sociodemographic, lifestyle, and comorbid factors also differed across the six sleep health patterns (Additional file 1: Table S7).

**Fig. 3:**
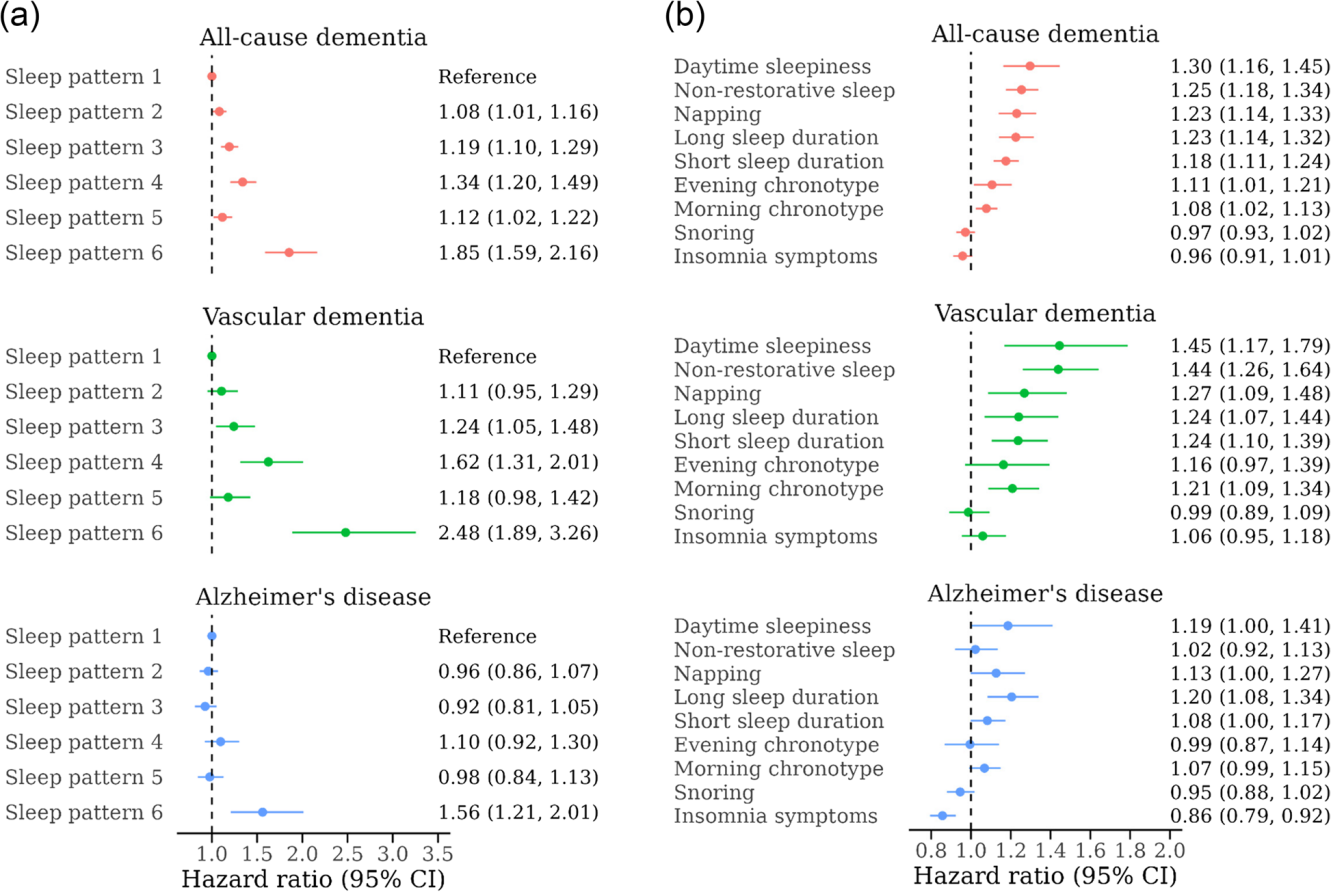
Associations of multi-dimensional sleep health with risk for all-cause dementia, vascular dementia, and Alzheimer’s disease among participants aged 50 years or older in the UK Biobank (2006–2022). **a** Multi-dimensional sleep health patterns identified by the latent class analysis. The sample size (%) was 209,918 (67.0%) for sleep pattern 1, 37,617 (12.0%) for pattern 2, 33,112 (10.6%) for pattern 3, 15,031 (4.8%) for pattern 4, 14,269 (4.6%) for pattern 5, and 3247 (1.0%) for pattern 6. **b** Individual sleep health factors. For short and long sleep duration, the reference group was medium sleep duration. For evening and morning chronotype, the reference group was intermediate chronotype. All estimates were adjusted for age (in months), sex, race/ethnicity, Townsend deprivation index, education (in years), employment status, family history of dementia, smoking status, alcohol consumption, physical activity, and body mass index

Compared with the relatively healthy sleep pattern, the other five sleep patterns were associated with significantly increased risk for all-cause dementia (Fig. 3a and Additional file 1: Fig. S1). The multivariable-adjusted HR (95% CI) compared with sleep pattern 1 was 1.08 (1.01, 1.16) for pattern 2, 1.19 (1.10, 1.29) for pattern 3, 1.34 (1.20, 1.49) for pattern 4, 1.12 (1.02, 1.22) for pattern 5, and 1.85 (1.59, 2.16) for pattern 6. A similar pattern of association was observed for VaD, with consistently stronger association estimates compared to those for all-cause dementia (range of comparable HRs for VaD: 1.11–2.48). However, only severely disturbed sleep was associated with increased AD risk. Compared with sleep pattern 1, sleep pattern 6 was associated with 56% higher AD risk (95% CI: 1.21, 2.01), whereas sleep patterns 2–5 showed no association with AD risk.

### Secondary analyses

SHS and sleep health clusters were strongly associated with clinically diagnosed sleep disorders, both at baseline and during follow-up (Additional file 1: Tables S8 and S9). For example, compared with participants with SHS of 6–7, those with SHS of 0–2 had 341% higher odds (95% CI: 3.88, 4.99) for prevalent sleep disorders at baseline and 4.59-fold increased risk (95%: 4.11, 5.12) of developing clinically diagnosed sleep disorders over follow-up. In the secondary analysis evaluating individual sleep health factors (Fig. 3b), daytime sleepiness, non-restorative sleep, napping, short/long sleep duration, and morning/evening chronotype were associated with 8–30% higher all-cause dementia risk and 16–45% higher VaD risk. The associations for AD were weaker and less consistent, and only daytime sleepiness, napping, and short/long sleep duration were linked to 8–20% higher AD risk. Insomnia symptoms and snoring, when examined individually, were not associated with increased risk for either all-cause dementia or dementia subtypes. In the competing risks analysis, poor sleep health was significantly associated with increased risk of premature death prior to dementia onset (Additional file 1: Table S10). However, accounting for these non-dementia-related deaths as competing events did not alter the associations of SHS or multi-dimensional sleep health patterns with all-cause dementia risk.

## Discussion

In this prospective study, multi-dimensional sleep health, either assessed by a simple SHS or characterized by sleep clusters, was associated with incident dementia risk after accounting for a wide range of covariates. SHS showed dose–response relationships with risks of all-cause dementia and VaD, whereas only the lowest SHS was associated with an increased AD risk. Similarly, compared with the healthy sleep pattern, increased risks for all-cause dementia and VaD were observed for all other five identified sleep health patterns, and only the severely disturbed sleep pattern was associated with higher AD risk. Our findings highlight the importance of adopting a multi-dimensional approach to assessing sleep health, which revealed heterogenous associations with dementia outcomes.

Experimental studies provide mechanistic insights into how sleep disturbances may accelerate neurodegenerative progression and dementia development. Healthy sleep may mitigate dementia pathology by strengthening existing neural connections, facilitating glymphatic clearance of waste products to reduce β-amyloid deposition, and supporting overall vascular health [25]. Conversely, sleep disorders, such as insomnia and sleep apnea, may lead to sleep fragmentation and cerebral hypoxia, enhancing neuroinflammation and damaging brain regions responsible for cognitive functions [26, 27]. Despite compelling experimental evidence, a recent Lancet Commission report does not include sleep health in the life-course model of 14 modifiable risk factors for dementia prevention, citing “not enough consistent evidence” from human studies [28].

One possible reason for the lack of consistent evidence in previous studies is the predominant focus on individual sleep dimensions without considering the complex relationships among different sleep domains and the heterogeneity in sleep health phenotypes. As supported by our SHS analysis, a greater number of unhealthy sleep factors was associated with higher dementia risk in a dose–response fashion. While many unhealthy sleep factors tend to co-occur, the degree of clustering can vary substantially based on age, sex, and other characteristics of the study sample. Therefore, examining individual sleep health factors without accounting for other sleep-related variables may lead to considerable variations in the direction and strength of associations across studies.

The importance of considering heterogeneity in sleep health phenotypes was further underscored by the LCA results. For example, we identified two sleep health patterns characterized by insomnia with short sleep duration (patterns 2 and 4). Although these patterns, based on self-reported short sleep duration, do not precisely align with the previously established insomnia with objective short sleep duration phenotype [13, 14], they exhibited distinct profiles regarding other sleep health factors. Pattern 2 was characterized by a high prevalence of morning chronotype without reported non-restorative sleep, whereas pattern 4 was characterized by a high prevalence of evening chronotype and the presence of non-restorative sleep. Further, these two patterns were associated with varying levels of dementia risk—an 8% increased risk for pattern 2 and a 34% increased risk for pattern 4, compared with pattern 1. Coupled with the analysis of individual sleep factors which showed no overall association with insomnia symptoms, these findings suggest that insomnia symptoms were linked to increased dementia risk only when accompanied by other adverse sleep factors (e.g., short sleep duration, non-restorative sleep). Insomnia symptoms alone seemed to have little association with dementia risk. Similar heterogeneity was also observed for snoring. While snoring alone was not associated with dementia risk, it was linked to increased risk in the presence of daytime sleepiness or napping (pattern 5). Pattern 5 may represent the “excessively sleepy sleep apnea” phenotype, which has been associated with increased cardiovascular disease risk yet remains frequently underdiagnosed in clinical settings [29–31]. This may also explain the stronger associations observed for VaD, given the well-established role of sleep apnea in vascular pathologies implicated in dementia, including systemic inflammation, glucose dysregulation, and hypertension [32–35].

Of note, a recent UK biobank study reported lower dementia risk comparing morning versus evening chronotypes [4]. The dichotomous classification of chronotype ignores the majority of individuals who fall into the intermediate category along the continuous spectrum [36]. Our results suggest that the lower dementia risk previously associated with morning chronotypes was actually driven by the intermediate type who reported “more morning than evening.” In fact, individuals who identified as “definitely morning” were at higher risk for dementia, particularly VaD, compared with those who reported being “more morning” or “more evening.” As the natural aging process is known to advance the circadian phase towards more morningness [37], these observations suggest that some morning chronotypes in older individuals may be a marker for accelerated aging, possibly contributing to neurodegeneration and dementia development. Future studies are needed to further subtype chronotype phenotypes to better understand the underlying genetic and biological differences.

Our analysis suggests that both the SHS and the identified sleep health patterns were more strongly associated with risk of VaD than AD. The strong associations with VaD may be attributed to the well-established impact of sleep health on vascular risk factors [38], although adjusting for multiple cardiovascular risk factors only modestly attenuated the associations. However, this does not account for the weaker associations observed for an AD diagnosis, where there is often co-occurring vascular pathology [39]. It is possible that the one-time sleep assessment conducted on average in the seventh decade may not be the most relevant measure for AD, which typically involves decades of amyloid and tau aggregation before the clinical onset of symptoms. Additionally, as subtype information was not available for all incident dementia cases and subject to measurement error, both selection bias and potential misclassification may have influenced the observed associations.

Self-reported sleep assessment is often the first step in clinical evaluations for risk assessment, diagnosis, and potential interventions. The heterogeneity in self-reported sleep health phenotypes, as shown in the current study, emphasizes the need for a multi-dimensional approach to assessing sleep health in order to more accurately identify inter-individual differences in susceptibility to sleep disturbances. For example, while sleep pattern 1 represented the healthiest sleep group with the lowest dementia risk, it still exhibited several unfavorable sleep health factors—39.8% reported loud snoring, 17.5% reported insomnia symptoms, 10.5% reported short sleep duration, and 7.8% reported long sleep duration, but almost none reported non-restorative sleep, napping, or sleepiness. It is unclear what factors may account for this divergence between nighttime symptoms and daytime functioning. Possible explanations include effects of the chronicity or severity of the sleep problems, or other genetic, socioenvironmental, or biological factors that influence “resilience” or vulnerability to sleep disruption. Nevertheless, our data support the importance in considering the effects of sleep disruption on daytime function when assessing dementia risk factors.

There are some issues that should be accounted for when interpreting the results. First, each sleep health dimension was assessed by a single-item question, which did not adequately reflect the severity, symptom heterogeneity, or chronicity of the underlying sleep disturbances. Also, these assessments did not encompass other important sleep health dimensions commonly measured by objective methods, such as sleep regularity [40–42], slow-wave sleep [8, 43], and hypoxic burden [44–46], which have been linked to risks of cardiometabolic disease and dementia. Nonetheless, the multi-dimensional sleep health patterns derived from these brief assessments showed strong associations with both prevalent and incident clinically diagnosed sleep disorders. Second, our analysis relied on a single sleep assessment conducted in middle age. While this one-time assessment has demonstrated moderate stability over time, it did not fully capture sleep health in early life or changes over time, which should be investigated in future studies [47, 48]. Additionally, the use of prevalent sleep health exposure has limited implications for interventions due to lack of information on the initiation and duration of sleep problems, and may potentially introduce selection bias and improper confounder adjustment [49]. Third, misclassification of dementia outcomes as indicated by the validation study [20], as well as missing dementia diagnoses or subtype information due to incomplete linkage to primary care data, may introduce bias into the associations in either direction. Fourth, despite the many strengths of LCA, this data-driven approach may face challenges in reproducibility when applied to different populations, owing to its probabilistic nature and model complexity. However, several sleep health patterns identified by our LCA were consistent with established sleep phenotypes, supporting its generalizability. Fifth, our observational study does not provide evidence of causality. Several Mendelian randomization studies reported weak evidence for a causal role of sleep traits in dementia [4, 5, 50–52]. However, these Mendelian randomization studies, along with the associated genome-wide association studies, share a key limitation: they focus on a single sleep factor rather than considering a multi-dimensional sleep health pattern. Finally, the volunteer-based sampling of the UK Biobank, which overrepresents healthier white participants with higher socioeconomic status, may bias the observed associations and limit their extrapolation to the general UK population [53].

## Conclusions

Poor sleep health assessed through multi-dimensional approaches was associated with higher dementia risk. Considerable heterogeneity exists with regard to sleep health patterns as well as their associations with dementia outcomes. Identifying the specific sleep health patterns linked to dementia risk could help pinpoint high-risk populations and guide the development of more targeted interventions.

## Supplementary information

Additional file 1: Tables S1–S10 and Fig. S1. Table S1 Assessment of self-reported sleep health factors. Table S2 Data fields for algorithmically defined dementia outcomes in the UK Biobank. Table S3 Associations of the multi-dimensional sleep health score with dementia risk excluding cases diagnosed in the first 5 years of follow-up. Table S4 Associations of the multi-dimensional sleep health score with dementia risk by age. Table S5 Associations of the multi-dimensional sleep health score with dementia risk by sex. Table S6 Associations of the multi-dimensional sleep health score with dementia risk by the polygenic risk score for Alzheimer’s disease. Table S7 Baseline characteristics by multi-dimensional sleep health patterns. Table S8 Cross-sectional associations of the multi-dimensional sleep health score and sleep health patterns with prevalent clinical sleep disorders. Table S9 Prospective associations of the multi-dimensional sleep health score and sleep health patterns with incident clinical sleep disorders. Table S10 Competing risks analysis. Fig. S1 Kaplan–Meier curves.

## Data Availability

The datasets generated/and or analyzed in the current study will be made available for researchers who apply to use the UK Biobank data set by registering and applying at http://www.ukbiobank.ac.uk/register-apply.

## Abbreviations

AD: Alzheimer’s disease
HR: Hazard ratio
ICC: Intraclass correlation coefficient
ICD-10: International Classification of Diseases 10th revision
LCA: Latent class analysis
PPV: Positive predicted value
SHS: Sleep health score
VaD: Vascular dementia
