# Supplementary material for: Multi-dimensional sleep health and dementia risk: a prospective study in the UK Biobank
Source: BMC Med. 2025 Jul 7;23:410. doi: 10.1186/s12916-025-04251-3 (PMC12235804; doi:10.1186/s12916-025-04251-3)

| Table S1. Assessment of self-reported sleep health factors^1^ | | | |
| --- | --- | --- | --- |
| Sleep health dimensions | Data field ID | Assessment questions | Responses and categorization^2^ |
| Sleep duration^3^ | 1160 | About how many hours sleep do you get in every 24 hours? (please include naps) | <7 hours (short) versus 7-8 hours (medium) versus >8 hours (long) |
| Insomnia symptoms | 1200 | Do you have trouble falling asleep at night or do you wake up in the middle of the night? | "Usually" versus "Sometimes", "Never/rarely" |
| Non-restorative sleep | 1170 | On an average day, how easy do you find getting up in the morning? | "Not at all easy", "Not very easy" versus "Fairly easy", "Very easy" |
| Daytime sleepiness | 1220 | How likely are you to doze off or fall asleep during the daytime when you don't mean to? (e.g., when working, reading or driving) | "Often" versus "Sometimes", "Never/rarely" |
| Snoring | 1210 | Does your partner or a close relative or friend complain about your snoring? | "Yes" versus "No" |
| Napping | 1190 | Do you have a nap during the day? | "Usually" versus "Sometimes", "Never/rarely" |
| Chronotype | 1180 | Do you consider yourself to be? | "Definitely a 'morning' person" (morning chronotype) versus "More a 'morning' than 'evening' person", "More an 'evening' than 'morning' person" (intermediate chronotype) versus "Definitely an ‘evening' person (evening chronotype) |
| ^1^Link to UK Biobank Showcase: <https://biobank.ndph.ox.ac.uk/showcase/label.cgi?id=100057> | | | |
| ^2^Participants who selected responses such as “prefer not to answer”, “do not know” or who skipped the question were classified as having missing data for that item and were excluded from the analysis | | | |
| ^3^Participants who reported sleep duration <3 hours or >14 hours were excluded from the analysis | | | |

| Table S2. Data fields for algorithmically-defined dementia outcomes in the UK Biobank | | | |
| --- | --- | --- | --- |
| Outcome | Description | Data-Field | Link |
|  |  |  |  |
| All-cause dementia | Date of all cause dementia report | 42018 | <https://biobank.ndph.ox.ac.uk/ukb/field.cgi?id=42018> |
| Alzheimer's disease | Date of alzheimer's disease report | 42020 | <https://biobank.ndph.ox.ac.uk/ukb/field.cgi?id=42020> |
| Vascular dementia | Date of vascular dementia report | 42022 | <https://biobank.ndph.ox.ac.uk/ukb/field.cgi?id=42022> |
|  |  |  |  |

| Table S3. Associations of the multi-dimensional sleep health score with risk of all-cause dementia, vascular dementia, and Alzheimer's disease in the UK Biobank, excluding cases diagnosed in the first 5 years of follow-up | | | | | |
| --- | --- | --- | --- | --- | --- |
|  | Multi-dimensional sleep health score | | | |  |
|  | 6 - 7  (best sleep) | 5 | 3 - 4 | 0-2  (worst sleep) | p for trend |
| All-cause dementia |  |  |  |  |  |
| Cases | 3221 | 1956 | 1568 | 157 |  |
| Person-years | 2102972 | 1162827 | 826436 | 52225 |  |
| Rate per 1,0000 person-years | 15.3 | 16.8 | 19.0 | 30.1 |  |
| Model 1^1^ | 1.00 (ref) | 1.08 (1.02, 1.14) | 1.21 (1.14, 1.29) | 1.75 (1.49, 2.06) | <0.0001 |
| Model 2^2^ | 1.00 (ref) | 1.07 (1.01, 1.13) | 1.18 (1.11, 1.25) | 1.64 (1.40, 1.93) | <0.0001 |
| Model 3^3^ | 1.00 (ref) | 1.05 (0.98, 1.12) | 1.13 (1.05, 1.21) | 1.43 (1.18, 1.75) | <0.0001 |
|  |  |  |  |  |  |
| Vascular dementia |  |  |  |  |  |
| Cases | 626 | 440 | 380 | 45 |  |
| Person-years | 2102972 | 1162827 | 826436 | 52225 |  |
| Rate per 1,0000 person-years | 3.0 | 3.8 | 4.6 | 8.6 |  |
| Model 1^1^ | 1.00 (ref) | 1.23 (1.09, 1.39) | 1.47 (1.29, 1.67) | 2.39 (1.76, 3.25) | <0.0001 |
| Model 2^2^ | 1.00 (ref) | 1.18 (1.05, 1.34) | 1.35 (1.19, 1.54) | 1.98 (1.45, 2.69) | <0.0001 |
| Model 3^3^ | 1.00 (ref) | 1.14 (0.99, 1.31) | 1.31 (1.12, 1.52) | 1.53 (1.04, 2.25) | <0.0001 |
|  |  |  |  |  |  |
| Alzheimer's disease |  |  |  |  |  |
| Cases | 1594 | 853 | 626 | 58 |  |
| Person-years | 2102972 | 1162827 | 826436 | 52225 |  |
| Rate per 1,0000 person-years | 7.6 | 7.3 | 7.6 | 11.1 |  |
| Model 1^1^ | 1.00 (ref) | 0.96 (0.88, 1.04) | 0.98 (0.90, 1.08) | 1.36 (1.05, 1.77) | 0.95 |
| Model 2^2^ | 1.00 (ref) | 0.96 (0.88, 1.04) | 0.98 (0.89, 1.08) | 1.37 (1.05, 1.78) | 0.99 |
| Model 3^3^ | 1.00 (ref) | 0.94 (0.86, 1.04) | 0.91 (0.82, 1.02) | 1.19 (0.86, 1.65) | 0.35 |
|  |  |  |  |  |  |
| ^1^Adjusted for age in months, sex, race/ethnicity, Townsend deprivation index, education in years, employment status and family history of dementia | | | | | |
| ^2^Model 1 + adjusted for smoking status, alcohol consumption, physical activity, and body mass index | | | | | |
| ^3^Model 2 + adjusted for total cholesterol, HDL cholesterol, systolic blood pressure, diastolic blood pressure, HbA1c and depression | | | | | |

| Table S4. Associations of the multi-dimensional sleep health score with risk of all-cause dementia, vascular dementia, and Alzheimer’s disease by age | | | | | |
| --- | --- | --- | --- | --- | --- |
|  | Multi-dimensional sleep health score | | | |  |
|  | 6 - 7  (best sleep) | 5 | 3 - 4 | 0-2  (worst sleep) | p for trend |
| All-cause dementia |  |  |  |  |  |
| ≥60 years | 1.00 (ref) | 1.05 (0.99, 1.11) | 1.14 (1.07, 1.21) | 1.60 (1.35, 1.89) | <0.0001 |
| <60 years | 1.00 (ref) | 1.25 (1.06, 1.48) | 1.49 (1.26, 1.78) | 2.75 (1.96, 3.85) | <0.0001 |
| P for interaction | 0.02 | | | | |
|  |  |  |  |  |  |
| Vascular dementia |  |  |  |  |  |
| ≥60 years | 1.00 (ref) | 1.18 (1.04, 1.33) | 1.30 (1.14, 1.48) | 1.82 (1.31, 2.51) | <0.0001 |
| <60 years | 1.00 (ref) | 1.19 (0.75, 1.90) | 2.17 (1.43, 3.31) | 4.89 (2.55, 9.37) | <0.0001 |
| P for interaction | 0.001 | | | | |
|  |  |  |  |  |  |
| Alzheimer's disease |  |  |  |  |  |
| ≥60 years | 1.00 (ref) | 0.94 (0.86, 1.02) | 0.97 (0.88, 1.06) | 1.47 (1.13, 1.90) | 0.99 |
| <60 years | 1.00 (ref) | 1.11 (0.85, 1.45) | 1.07 (0.80, 1.44) | 2.18 (1.18, 4.02) | 0.07 |
| P for interaction | 0.05 | | | | |
|  |  |  |  |  |  |
| ^1^Adjusted for age in months, sex, race/ethnicity, Townsend deprivation index, education in years, employment status, family history of dementia, smoking status, alcohol consumption, physical activity, and body mass index | | | | | |

| Table S5. Associations of the multi-dimensional sleep health score with risk of all-cause dementia, vascular dementia, and Alzheimer's disease by sex | | | | | |
| --- | --- | --- | --- | --- | --- |
|  | Multi-dimensional sleep health score | | | |  |
|  | 6 - 7  (best sleep) | 5 | 3 - 4 | 0-2  (worst sleep) | p for trend |
| All-cause dementia |  |  |  |  |  |
| Men | 1.00 (ref) | 1.04 (0.96, 1.12) | 1.17 (1.08, 1.27) | 1.50 (1.22, 1.83) | <0.0001 |
| Women | 1.00 (ref) | 1.10 (1.02, 1.19) | 1.17 (1.07, 1.27) | 2.19 (1.75, 2.73) | <0.0001 |
| P for interaction | 0.47 | | | | |
|  |  |  |  |  |  |
| Vascular dementia |  |  |  |  |  |
| Men | 1.00 (ref) | 1.06 (0.91, 1.23) | 1.24 (1.05, 1.45) | 1.59 (1.09, 2.30) | 0.003 |
| Women | 1.00 (ref) | 1.36 (1.13, 1.63) | 1.54 (1.27, 1.87) | 3.37 (2.19, 5.19) | <0.0001 |
| P for interaction | 0.03 | | | | |
|  |  |  |  |  |  |
| Alzheimer's disease |  |  |  |  |  |
| Men | 1.00 (ref) | 0.87 (0.77, 0.97) | 0.92 (0.80, 1.05) | 1.39 (1.01, 1.93) | 0.40 |
| Women | 1.00 (ref) | 1.02 (0.92, 1.14) | 1.02 (0.90, 1.16) | 1.74 (1.23, 2.48) | 0.14 |
| P for interaction | 0.10 | | | | |
|  |  |  |  |  |  |
| ^1^Adjusted for age in months, race/ethnicity, Townsend deprivation index, education in years, employment status, family history of dementia, smoking status, alcohol consumption, physical activity, and body mass index | | | | | |

| Table S6. Associations of the multi-dimensional sleep health score with risk of all-cause dementia, vascular dementia, and Alzheimer's disease, stratified by the tertiles of the polygenic risk score for Alzheimer's disease | | | | | |
| --- | --- | --- | --- | --- | --- |
|  | Multi-dimensional sleep health score | | | |  |
|  | 6 - 7  (best sleep) | 5 | 3 - 4 | 0-2  (worst sleep) | p for trend |
| All-cause dementia |  |  |  |  |  |
| PRS tertile 1 | 1.00 (ref) | 1.19 (1.05, 1.35) | 1.30 (1.13, 1.49) | 2.21 (1.63, 3.02) | <0.0001 |
| PRS tertile 2 | 1.00 (ref) | 1.26 (1.13, 1.41) | 1.36 (1.20, 1.53) | 2.35 (1.78, 3.11) | <0.0001 |
| PRS tertile 3 | 1.00 (ref) | 0.98 (0.91, 1.06) | 1.11 (1.03, 1.20) | 1.47 (1.17, 1.85) | 0.0007 |
| P for interaction | <0.0001 | | | | |
|  |  |  |  |  |  |
| Vascular dementia |  |  |  |  |  |
| PRS tertile 1 | 1.00 (ref) | 1.42 (1.09, 1.86) | 1.64 (1.24, 2.17) | 2.71 (1.50, 4.87) | <0.0001 |
| PRS tertile 2 | 1.00 (ref) | 1.35 (1.08, 1.71) | 1.41 (1.10, 1.82) | 3.14 (1.88, 5.22) | <0.0001 |
| PRS tertile 3 | 1.00 (ref) | 1.05 (0.89, 1.23) | 1.26 (1.06, 1.50) | 1.79 (1.16, 2.77) | 0.002 |
| P for interaction | 0.001 | | | | |
|  |  |  |  |  |  |
| Alzheimer's disease |  |  |  |  |  |
| PRS tertile 1 | 1.00 (ref) | 1.13 (0.91, 1.39) | 1.09 (0.86, 1.38) | 2.67 (1.66, 4.31) | 0.10 |
| PRS tertile 2 | 1.00 (ref) | 1.11 (0.93, 1.33) | 1.17 (0.96, 1.42) | 1.71 (1.03, 2.85) | 0.02 |
| PRS tertile 3 | 1.00 (ref) | 0.88 (0.79, 0.97) | 0.92 (0.82, 1.04) | 1.34 (0.96, 1.88) | 0.49 |
| P for interaction | <0.0001 | | | | |
|  |  |  |  |  |  |
| ^1^Adjusted for age in months, sex, race/ethnicity, Townsend deprivation index, education in years, employment status, family history of dementia, smoking status, alcohol consumption, physical activity, and body mass index, as well as the first four genetic principal components | | | | | |

| Table S7. Baseline characteristics by multi-dimensional sleep health patterns in the UK Biobank | | | | | | |
| --- | --- | --- | --- | --- | --- | --- |
|  | Sleep patterns | | | | | |
|  | 1 | 2 | 3 | 4 | 5 | 6 |
| N | 209918 | 37671 | 33112 | 15031 | 14269 | 3247 |
| Age, years | 60.7 (5.4) | 60.7 (5.4) | 59.1 (5.5) | 59.1 (5.4) | 62.5 (5.3) | 61.0 (5.6) |
| Men, % | 47.3 | 37.5 | 34.2 | 34.9 | 68.9 | 57.1 |
| White, % | 96.5 | 95.7 | 95.5 | 92.2 | 95.4 | 92.6 |
| Townsend deprivation index | -1.7 (2.9) | -1.3 (3.1) | -1.4 (3.0) | -0.8 (3.3) | -1.3 (3.0) | -0.1 (3.4) |
| Education, years | 14.7 (5.2) | 13.7 (5.3) | 15.1 (5.0) | 14.4 (5.2) | 14.3 (5.4) | 13.1 (5.5) |
| Employment status |  |  |  |  |  |  |
| Employed | 49.2 | 47.9 | 53.8 | 54.0 | 36.4 | 25.1 |
| Retired | 44.6 | 43.4 | 34.2 | 30.9 | 55.2 | 48.1 |
| Unemployed/unable to work | 6.2 | 8.6 | 12.0 | 15.1 | 8.4 | 26.8 |
| Family history of dementia, % | 13.7 | 14.0 | 14.1 | 14.0 | 13.4 | 13.4 |
| Smoking status, % |  |  |  |  |  |  |
| Never | 53.7 | 52.3 | 52.2 | 49.0 | 45.3 | 41.9 |
| Current | 8.3 | 8.9 | 11.9 | 15.3 | 11.5 | 17.4 |
| Former | 38.0 | 38.8 | 35.9 | 35.7 | 43.2 | 40.7 |
| Alcohol drinking, % |  |  |  |  |  |  |
| Never/special occasion only | 17.2 | 22.4 | 20.6 | 26.2 | 20.6 | 35.0 |
| 1-3 per month | 10.0 | 10.7 | 11.1 | 12.0 | 8.9 | 11.6 |
| 1-4 per week | 50.0 | 46.4 | 46.4 | 43.4 | 44.1 | 36.0 |
| Daily/almost daily | 22.8 | 20.4 | 22.0 | 18.3 | 26.4 | 17.4 |
| Physical activity, MET-hrs/week | 45.6 (43.9) | 48.4 (47.6) | 35.1 (37.7) | 37.0 (42.2) | 47.2 (48.2) | 32.0 (41.1) |
| Body mass index, kg/m2 | 27.3 (4.5) | 27.7 (4.9) | 27.5 (5.0) | 28.3 (5.4) | 28.3 (4.8) | 30.3 (6.3) |
| Systolic blood pressure, mmHg | 141.0 (18.6) | 140.3 (18.6) | 137.3 (18.6) | 137.8 (18.3) | 142.6 (18.6) | 139.4 (19.0) |
| Diastolic blood pressure, mmHg | 82.7 (10.0) | 82.3 (10.0) | 81.8 (10.2) | 82.1 (10.1) | 83.0 (10.2) | 82.3 (10.5) |
| Total cholesterol, mg/dL | 223.5 (44.9) | 223.5 (45.3) | 224.4 (45.3) | 222.7 (45.7) | 212.2 (47.1) | 207.7 (49.6) |
| HDL cholesterol, mg/dL | 56.8 (15.0) | 57.7 (15.3) | 57.4 (15.3) | 56.4 (15.2) | 51.9 (14.1) | 50.1 (13.7) |
| HbA1c, mmol/mol | 36.4 (6.4) | 36.8 (6.7) | 36.6 (7.0) | 37.2 (7.4) | 38.1 (8.5) | 40.0 (10.7) |
| Depression^1^ | 31.9 | 42.5 | 51.0 | 55.5 | 37.0 | 64.2 |
| Polygenic risk score for AD | 0.06 (1.00) | 0.05 (0.99) | 0.05 (0.99) | 0.03 (0.99) | 0.03 (0.98) | 0.02 (0.99) |
| Sleep health |  |  |  |  |  |  |
| Sleep duration, % |  |  |  |  |  |  |
| Short (<7 hours) | 10.5 | 98.4 | 0.0 | 99.9 | 8.3 | 40.9 |
| Medium (7-8 hours) | 81.7 | 1.6 | 85.3 | 0.1 | 67.9 | 21.4 |
| Long (>8 hours) | 7.8 | 0.0 | 14.7 | 0.0 | 23.8 | 37.7 |
| Insomnia symptoms, % | 17.5 | 80.8 | 31.6 | 63.3 | 26.5 | 72.6 |
| Non-restorative sleep, % | 0.0 | 0.0 | 100.0 | 82.6 | 0.0 | 75.6 |
| Snoring, % | 39.8 | 26.9 | 39.0 | 34.1 | 46.9 | 54.4 |
| Daytime sleepiness, % | 0.6 | 5.5 | 2.6 | 4.8 | 17.4 | 56.1 |
| Napping, % | 0.0 | 2.8 | 3.5 | 0.0 | 94.2 | 95.1 |
| Chronotype, % |  |  |  |  |  |  |
| Morning | 28.9 | 51.6 | 5.3 | 7.6 | 40.2 | 20.5 |
| Intermediate | 66.8 | 48.4 | 68.7 | 53.5 | 54.7 | 52.9 |
| Evening | 4.4 | 0.0 | 26.0 | 38.9 | 5.1 | 26.6 |
|  |  |  |  |  |  |  |
| ^1^Based on a broad depression definition if one or more of the following was met: Patient Health Questionnaire-2 (PHQ-2) score≥3 or self-reported clinical visits for depression | | | | | | |

| Table S8. Cross-sectional associations of the multi-dimensional sleep health score and sleep health patterns with prevalent clinical sleep disorders at baseline^1^ | | | | | |
| --- | --- | --- | --- | --- | --- |
|  | Cases | N | Model 1^2^ | Model 2^3^ | Model 3^4^ |
|  |  |  | Odds ratio (95% CI) | | |
| Sleep health score |  |  |  |  |  |
| 6-7 (best sleep) | 1990 | 158057 | 1.00 (ref) | 1.00 (ref) | 1.00 (ref) |
| 5 | 1845 | 87942 | 1.66 (1.56, 1.77) | 1.55 (1.46, 1.66) | 1.51 (1.41, 1.63) |
| 3-4 | 2259 | 63068 | 2.84 (2.67, 3.02) | 2.48 (2.33, 2.64) | 2.21 (2.06, 2.38) |
| 0-2 (worst sleep) | 325 | 4181 | 5.98 (5.28, 6.76) | 4.41 (3.88, 4.99) | 3.45 (2.97, 4.00) |
|  |  |  |  |  |  |
| Sleep health patterns | |  |  |  |  |
| Pattern 1 | 3129 | 209918 | 1.00 (ref) | 1.00 (ref) | 1.00 (ref) |
| Pattern 2 | 1237 | 37671 | 2.30 (2.15, 2.46) | 2.23 (2.08, 2.38) | 2.12 (1.96, 2.29) |
| Pattern 3 | 821 | 33112 | 1.75 (1.62, 1.89) | 1.66 (1.54, 1.80) | 1.43 (1.30, 1.56) |
| Pattern 4 | 629 | 15031 | 2.96 (2.70, 3.23) | 2.64 (2.41, 2.89) | 2.14 (1.92, 2.38) |
| Pattern 5 | 376 | 14269 | 1.60 (1.43, 1.78) | 1.47 (1.31, 1.64) | 1.27 (1.11, 1.44) |
| Pattern 6 | 227 | 3247 | 4.34 (3.75, 4.98) | 3.32 (2.86, 3.82) | 2.52 (2.11, 2.97) |
|  |  |  |  |  |  |
| ^1^Clinical sleep disorders were identified based on ICD codes including insomnia, hypersomnia, circadian rhythm sleep disorders, sleep apnea, narcolepsy, parasomnia, sleep-related movement disorders, and other/unspecified sleep disorders | | | | | |
| ^2^Adjusted for age in months, sex, race/ethnicity, Townsend deprivation index, education in years, employment status and family history of dementia | | | | | |
| ^3^Model 1 + adjusted for smoking status, alcohol consumption, physical activity and body mass index | | | | | |
| ^4^Model 2 + adjusted for total cholesterol, HDL cholesterol, systolic blood pressure, diastolic blood pressure, HbA1c and depression | | | | | |

| Table S9. Prospective associations of the multi-dimensional sleep health score and sleep health patterns with incident clinical sleep disorders^1^ | | | | | | |
| --- | --- | --- | --- | --- | --- | --- |
|  | Cases | Person-years | Rate per 10,000 person-years | Model 1^2^ | Model 2^3^ | Model 3^4^ |
|  |  |  |  | Hazard ratio (95% CI) | | |
| Sleep health score |  |  |  |  |  |  |
| 6-7 (best sleep) | 2053 | 2080984 | 9.9 | 1.00 (ref) | 1.00 (ref) | 1.00 (ref) |
| 5 | 2132 | 1135163 | 18.8 | 1.84 (1.73, 1.95) | 1.66 (1.57, 1.77) | 1.62 (1.51, 1.74) |
| 3-4 | 2615 | 787969 | 33.2 | 3.16 (2.99, 3.35) | 2.59 (2.44, 2.74) | 2.30 (2.15, 2.47) |
| 0-2 (worst sleep) | 405 | 46549 | 87.0 | 7.20 (6.46, 8.02) | 4.59 (4.11, 5.12) | 3.90 (3.43, 4.43) |
|  |  |  |  |  |  |  |
| Sleep health patterns |  |  |  |  |  |  |
| Pattern 1 | 3687 | 2747034 | 13.4 | 1.00 (ref) | 1.00 (ref) | 1.00 (ref) |
| Pattern 2 | 1107 | 478135 | 23.2 | 1.76 (1.64, 1.88) | 1.67 (1.56, 1.79) | 1.54 (1.42, 1.66) |
| Pattern 3 | 940 | 424976 | 22.1 | 1.73 (1.61, 1.86) | 1.62 (1.51, 1.74) | 1.45 (1.33, 1.58) |
| Pattern 4 | 610 | 186746 | 32.7 | 2.41 (2.21, 2.63) | 2.06 (1.88, 2.24) | 1.78 (1.60, 1.97) |
| Pattern 5 | 566 | 177860 | 31.8 | 2.01 (1.84, 2.20) | 1.78 (1.63, 1.94) | 1.65 (1.49, 1.83) |
| Pattern 6 | 295 | 35915 | 82.1 | 4.74 (4.20, 5.35) | 3.26 (2.88, 3.68) | 2.62 (2.27, 3.02) |
|  |  |  |  |  |  |  |
| ^1^Based on 306,829 participants after further excluding 6,419 with prevalent clinical sleep disorders at baseline. Clinical sleep disorders were identified based on ICD codes including insomnia, hypersomnia, circadian rhythm sleep disorders, sleep apnea, narcolepsy, parasomnia, sleep-related movement disorders, and other/unspecified sleep disorders | | | | | | |
| ^2^Adjusted for age in months, sex, race/ethnicity, Townsend deprivation index, education in years, employment status and family history of dementia | | | | | | |
| ^3^Model 1 + adjusted for smoking status, alcohol consumption, physical activity and body mass index | | | | | |  |
| ^4^Model 2 + adjusted for total cholesterol, HDL cholesterol, systolic blood pressure, diastolic blood pressure, HbA1c and depression | | | | | | |

| Table S10. Competing risks analysis for the associations of the multi-dimensional sleep health score and sleep health patterns with all-cause dementia risk and non-dementia-related mortality^1^ | | | | | |
| --- | --- | --- | --- | --- | --- |
|  | All-cause dementia | |  | Non-dementia-related mortality | |
|  | Cases | HR (95% CI)^2^ |  | Cases | HR (95% CI)^2^ |
|  |  |  |  | | |
| Sleep health score |  |  |  |  |  |
| 6-7 (best sleep) | 3463 | 1.00 (ref) |  | 13018 | 1.00 (ref) |
| 5 | 2113 | 1.06 (1.01, 1.12) |  | 8129 | 1.04 (1.01, 1.07) |
| 3-4 | 1697 | 1.15 (1.09, 1.22) |  | 6864 | 1.14 (1.10, 1.17) |
| 0-2 (worst sleep) | 185 | 1.64 (1.41, 1.91) |  | 756 | 1.43 (1.32, 1.54) |
|  |  |  |  |  |  |
| Sleep health patterns | |  |  |  |  |
| Pattern 1 | 4746 | 1.00 (ref) |  | 17943 | 1.00 (ref) |
| Pattern 2 | 958 | 1.08 (1.00, 1.15) |  | 3543 | 1.08 (1.04, 1.12) |
| Pattern 3 | 699 | 1.15 (1.06, 1.25) |  | 2977 | 1.15 (1.11, 1.20) |
| Pattern 4 | 369 | 1.28 (1.15, 1.43) |  | 1592 | 1.24 (1.18, 1.30) |
| Pattern 5 | 516 | 1.11 (1.01, 1.21) |  | 2033 | 1.16 (1.11, 1.22) |
| Pattern 6 | 170 | 1.70 (1.45, 1.99) |  | 679 | 1.54 (1.42, 1.66) |
|  |  |  |  |  |  |
| ^1^Deaths from causes unrelated to dementia were treated as competing events, and modeled using the Fine and Gray competing risks approach | | | | | |
| ^2^Adjusted for age in months, sex, race/ethnicity, Townsend deprivation index, education in years, employment status, family history of dementia, smoking status, alcohol consumption, physical activity and body mass index | | | | | |

Fig. S1. Estimated Kaplan-Meier curves of probability of remaining free of all-cause dementia with age, according to (A) the Sleep Health Score (SHS) and (B) the multi-dimensional sleep health patterns in the UK Biobank.

(A)


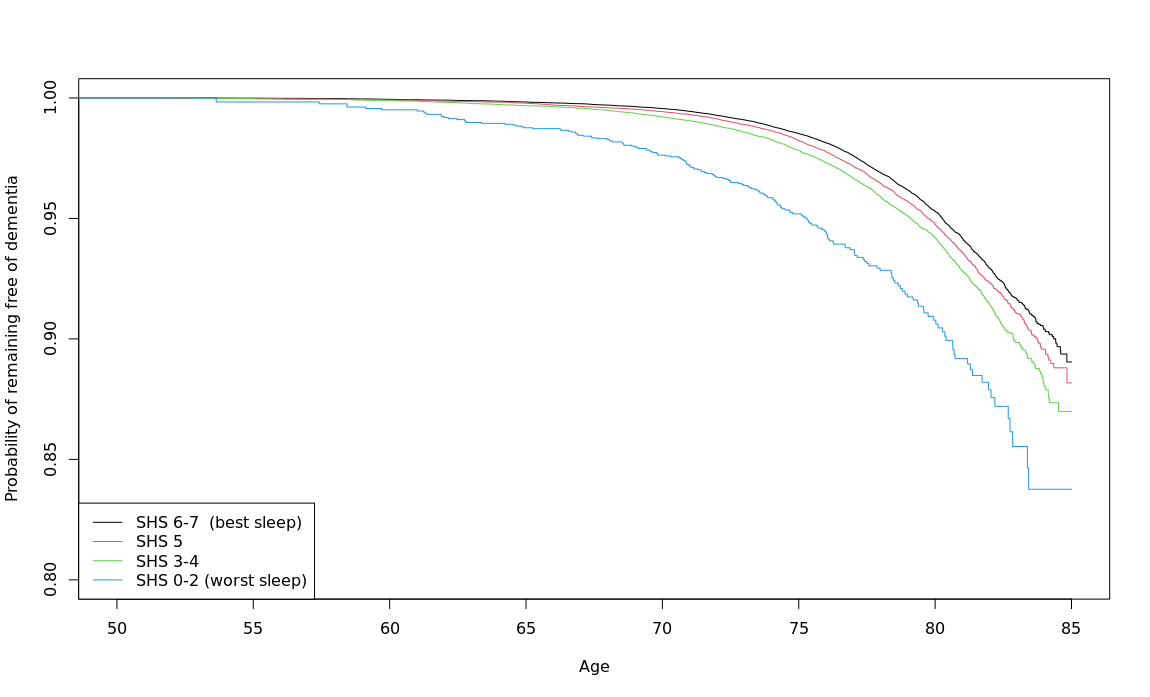


(B)


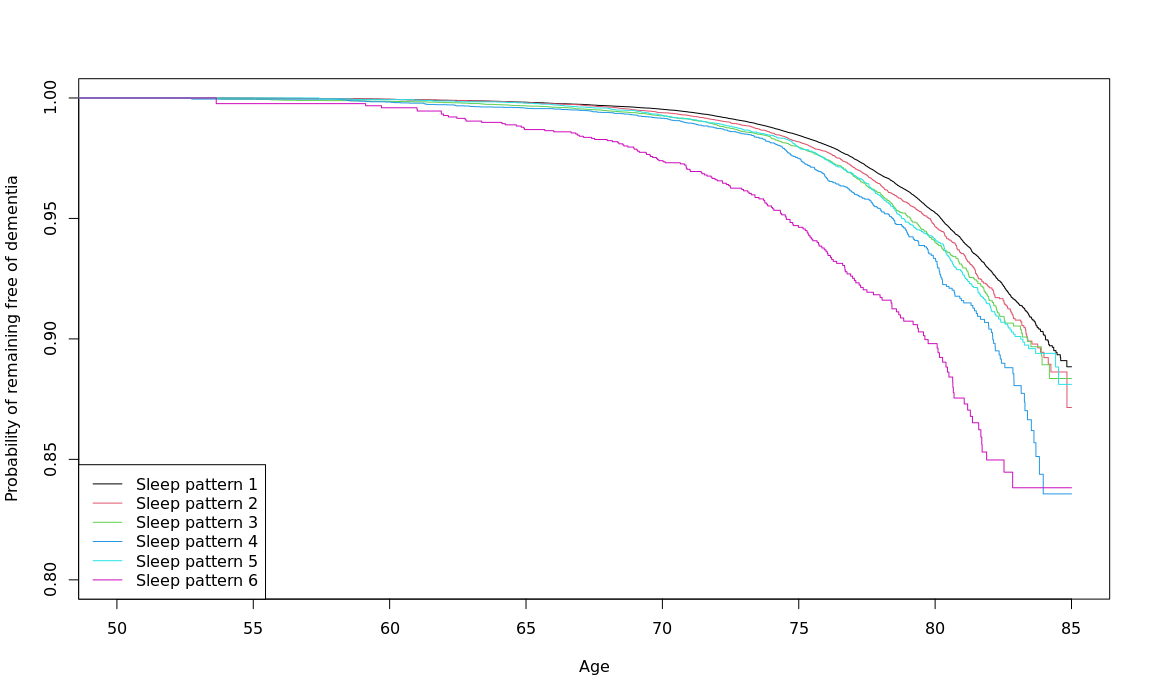

Supplement: Supplementary file 1 — Additional file 1: Tables S1–S10 and Fig. S1. Table S1 Assessment of self-reported sleep health factors. Table S2 Data fields for algorithmically defined dementia outcomes in the UK Biobank. Table S3 Associations of the multi-dimensional sleep health score with dementia risk excluding cases diagnosed in the first 5 years of follow-up. Table S4 Associations of the multi-dimensional sleep health score with dementia risk by age. Table S5 Associations of the multi-dimensional sleep health score with dementia risk by sex. Table S6 Associations of the multi-dimensional sleep health score with dementia risk by the polygenic risk score for Alzheimer’s disease. Table S7 Baseline characteristics by multi-dimensional sleep health patterns. Table S8 Cross-sectional associations of the multi-dimensional sleep health score and sleep health patterns with prevalent clinical sleep disorders. Table S9 Prospective associations of the multi-dimensional sleep health score and sleep health patterns with incident clinical sleep disorders. Table S10 Competing risks analysis. Fig. S1 Kaplan–Meier curves. [file 12916_2025_4251_MOESM1_ESM.docx]
